# Identification of Single Nucleotide Polymorphism in *TaSBEIII* and Development of KASP Marker Associated With Grain Weight in Wheat

**DOI:** 10.3389/fgene.2021.697294

**Published:** 2021-07-09

**Authors:** Ahsan Irshad, Huijun Guo, Shoaib Ur Rehman, Xueqing Wang, Jiayu Gu, Hongchun Xiong, Yongdun Xie, Linshu Zhao, Shirong Zhao, Chaojie Wang, Luxiang Liu

**Affiliations:** ^1^Institute of Crop Sciences, Chinese Academy of Agricultural Sciences, National Engineering Laboratory of Crop Molecular Breeding, National Center of Space Mutagenesis for Crop Improvement, Beijing, China; ^2^Institute of Plant Breeding and Biotechnology, Muhammad Nawaz Sharif University of Agriculture, Multan, Pakistan

**Keywords:** wheat, association analysis, KASP, *TaSBEIII*, polymorphisms, molecular marker

## Abstract

Manipulation of genes involved in starch synthesis could significantly affect wheat grain weight and yield. The starch-branching enzyme (SBE) catalyzes the formation of branch points by cleaving the α-1,4 linkage in polyglucans and reattaching the chain *via* an α-1,6 linkage. Three types of SBE isoforms (SBEI, SBEII, and SBEIII) exist in higher plants, with the number of SBE isoforms being species-specific. In this study, the coding sequence of the wheat *TaSBEIII* gene was amplified. After the multiple sequence alignment of *TaSBEIII* genome from 20 accessions in a wheat diversity panel, one SNP was observed in *TaSBEIII-A*, which formed the allelic marker *allele-T*. Based on this SNP at 294 bp (C/T), a KASP molecular marker was developed to distinguish allelic variation among the wheat genotypes for thousand grain weight (TGW). The results were validated using 262 accessions of mini core collection (MCC) from China, 153 from Pakistan, 53 from CIMMYT, and 17 diploid and 18 tetraploid genotypes. Association analysis between *TaSBEIII-A* allelic variation and agronomic traits found that *TaSBEIII-A* was associated with TGW in mini core collection of China (MCC). The accessions possessing *Allele-T* had higher TGW than those possessing *Allele-C*; thus, *Allele-T* was a favorable allelic variation. By analyzing the frequency of the favorable allelic variation *Allele-T* in MCC, it increased from pre-1950 (25%) to the 1960s (45%) and increased continuously from 1960 to 1990 (80%). The results suggested that the KASP markers can be utilized in grain weight improvement, which ultimately improves wheat yield by marker-assisted selection in wheat breeding. The favorable allelic variation *allele-T* should be valuable in enhancing grain yield by improving the source and sink simultaneously. Furthermore, the newly developed KASP marker validated in different genetic backgrounds could be integrated into a breeding kit for screening high TGW wheat.

## Introduction

Wheat is the staple food for ∼33% of the population across the globe (Su et al., 2011). According to a worldwide survey, food demand will increase by 40% in the post-2020 era due to the rapid increase in global population (Su et al., 2011; Wang et al., 2019). To ensure this food security issue, there is a need to develop high-yield varieties through advanced molecular breeding (Wang et al., 2019). The valuable source in plants for energy and carbon is starch. The production of starch takes place in green leaves of plants during photosynthesis and surplus glucose is produced (Irshad et al., 2019). There is a relation of source and sink at the physiological level that helps to determine the yield of the crops (Rossi et al., 2015). Sink capacity is more important in wheat as compared to source so there is a need to explore the starch synthesis enzymes to accelerate the wheat yield through breeding (Hou et al., 2014). Starch mainly consists of amylose and amylopectin in which amylopectin contributed 75% in the starch granule (Pfister and Zeeman, 2016). The metabolism process of different enzymes [ADP-glucose pyrophosphorylases, starch synthases, starch-branching enzymes (SBEs), and starch-debranching enzymes] helps in the formation of starch (Bertoft, 2017).

Metabolism of starch significantly affects the yield and quality of wheat because it accounts for about 65–80% of the grain endosperm (Xia et al., 2020). Functional changes in starch genes dramatically influence the starch content, amylose content, and other agronomic traits. One of the most important yield contributing trait in wheat is the thousand grain weight (TGW). Different starch synthesis genes played a role in TGW in wheat such as decreasing the expression of *GWD via* RNAi and significantly increasing the grain number per plant and TGW (Ral et al., 2012). Similarly, three haplotypes were detected in *TaSSIV-A*, and these haplotypes were associated with TGW. *Hap-2-1A* showed a significant difference from the other two haplotypes and had higher TGW (Irshad et al., 2019). It can be suggested that optimizing starch metabolism might improve the TGW, and its value can be increased with significant genetic improvement in wheat grain yield (Zheng et al., 2011).

The main function of SBE is the formation of branch points through cleavage of the polyglucan chains from α-1,4 linkage and reattachment of these polyglucan chains *via* α-1,6 linkage. Based on physiological and biochemical properties, SBE has three types of isoforms (SBEI, SBEII, and SBEIII) (Yan et al., 2009). SBEIIa and SBEIIb are two further subclades of SBEII. SBEI is present in wheat, rice, maize, and other plants except for *Arabidopsis* (Kang et al., 2013). Different research work has been done on SBE proteins especially on SBEI, SBEIIa, and SBEIIb (Stamova et al., 2009; Jeon et al., 2010). There is limited information about SBEIII due to the challenging tasks in isolation and purification of the coded protein of this gene (Yan et al., 2009). In wheat, the *TaSBEIII* CDS sequence, which consists of 3780 bp with an open reading frame of 2748 bp, was identified through the RACE method from common wheat, which reveals the existence of the *SBEIII* gene in common wheat. SBEIII has special characteristics based on the predicted protein of 916 amino acids with four highly conserved domains (Kang et al., 2013). The *SBEIII* gene is reported in many higher plants but there remains no information in lower plants (Han et al., 2007). Based on this information, it is depicted that *SBEIII* is different from *SBEI*, and when higher plants were separated from lower plants, then this gene could have arisen during the evolutionary process of gene divergence and duplication. The expression of *TaSBEIII* was consistent during grain filling period in wheat, giving the idea that its function is different from other SBE enzymes and its main function is the formation of A and B granules in the grains of wheat (Kang et al., 2013).

To accelerate the process of the wheat breeding program, the potential approach is the marker-assisted selection (MAS) (Zheng et al., 2017). Single nucleotide change and deletion or insertion in a nucleotide sequence is referred to as single nucleotide polymorphism (SNP) (Liu et al., 2012). SNP markers are more reliable as compared to other markers (RFLP, RAPD, AFLP, SSR, and ISSR) due to their high relative stability and high-throughput scoring (Wang et al., 2015). Similarly, it has been reported that SNPs are effective markers in fine mapping, association analysis, and functional marker development (Zhang et al., 2013; Qi et al., 2014). By using the SNPs in association analysis, it has been documented that it is an effective tool for the identification of relationship between the polymorphic site of target gene and quantitative traits. These analyses have been widely used in many crops such as in *Arabidopsis*, maize, rice, and wheat (Hayashi et al., 2004; Li et al., 2010, 2016; Nemri et al., 2010; Irshad et al., 2019). Different methodologies have been used in the development of markers for different genes such as high-resolution melting (HRM) and cleaved amplified polymorphism (CAPS) (Chen et al., 2014; Luo et al., 2014). However, due to some limitations, the utilization of these markers is limited because the HRM method needs multiple PCR cycles for their unique PCR product and CAPS markers also need special enzymes for special pair digestion. Due to these reasons, the breeder needs a friendly and high-throughput marker methodology for diverse breeding programs. The utilization of the newly developed Kompetitive Allele Specific PCR (KASP), which is a gel-free assay that has an allele-specific PCR, has addressed this issue. These markers are suitable for high-throughput genotyping of SNPs and also for insertion/deletion (He et al., 2014). Multiple alleles arise due to the presence of SNPs and InDels in the sequence of nucleotides (Rasheed et al., 2016). These SNPs contribute directly to the phenotypic variation, and this type of polymorphism is important for the development of functional markers. The efficiency of selection in wheat breeding and speed can be improved by conversion of the functional markers into KASP (Rasheed et al., 2018a). The *TaSBEIII* is present in chromosome seven in the A, B, and D genome and plays an important role in granule formation during the grain filling stage, but still, polymorphism of *TaSBEIII* is unclear in wheat.

**FIGURE 1:**
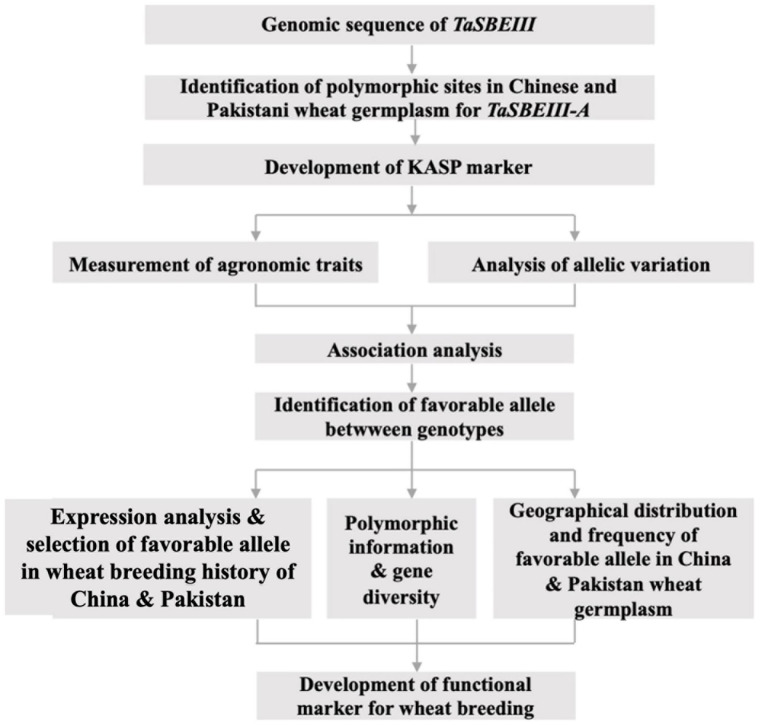
The schematic display of research work.

The main purposes of this study were to (a) identify the polymorphic site and development of the high-throughput KASP marker; (b) identify variation in favorable alleles; (c) identify the geographic distribution of the allelic variations in Chinese, CIMMYT, and Pakistani wheat germplasms; (d) identify gene diversity in diploid, tetraploid, and hexaploid wheat germplasm; and (e) analyze the association between polymorphic sites and phenotypic traits. The research work scheme is shown in Figure 1.

## Materials and Methods

### Plant Material and Morphological Data

A diversity panel of 20 accessions was selected, which consists of Chinese wheat landraces (7), Chinese modern wheat cultivars (7)m and wheat accessions from Pakistan (6) to detect the polymorphism in *TaSBEIII* (A, B, and D sub-genomes). The Chinese wheat mini core collection (MCC) consisted of 262 accessions representing more than 70% genetic diversity of wheat germplasm in China (Supplementary File 1; Hao et al., 2008), 153 accessions from Pakistan, 53 CIMMYT accessions, and 16 diploid and 19 tetraploid wheat accessions, which were used to evaluate the newly developed molecular markers and their distribution and frequency in different zones. The MCC collection was planted at the Chinese Academy of Agricultural Sciences (CAAS) experimental station in Beijing 2017–2018 (111.6°E, 33.8°N). The plants were grown with two replications, and each single-row plot was 4 m long with a 75-cm space between rows. The plant spacing within the row was 10 cm. Field management was done according to the normal agricultural practices. The data of yield-related traits (TGW, number of grains per spike, plant height, and spike length) were collected from the MCC 262 accessions and used in the present study. Grain numbers were calculated per spike with three replications and TGW was calculated after harvesting the crop by counting the 1,000 seeds from each accessions with three replications. Plant height and spike length were measured at the maturing stage of the crop with the help of a centimeter scale.

### Isolation of DNA, Amplification of PCR, Sequencing, and Alignment

DNA of all the accessions was extracted by using the CTAB method (Gawel and Jarret, 1991). The purification of DNA was done by “RNase A” treatment and isoamyl alcohol precipitation (Sambrook, 1987). Agarose gel was used to check the quality and quantity of the DNA. The gene primer *TaSBEIII-F/R* was used to amplify all three genomes simultaneously for the *TaSBEIII* gene covering the CDS sequence (Table 1). PCR reaction was carried out in a total volume of 15 μl as initial denaturation at 95°C for 3 min, followed by 32 cycles at 95°C for 30 s, annealing at 58°C for 30 s, and extension at 72°C for 3 min, with a final extension at 72°C for 10 min. Agarose gel of ∼1% was used to resolve the PCR product by electrophoresis. The resulting PCR fragment was directly sequenced from both directions by a commercial company (Sangon Biotech Co., Ltd., China) to find out the polymorphisms. MEGA software^1^ was used to align this 20-genotypes sequence, and Chinese Spring sequence was used as a reference sequence that was downloaded from the NCBI database with accession no. JQ34619.

**TABLE 1:** Primers used in the experiments.

**Primer name**	**Primer sequence 5 to 3**	**Purpose**
*TaSBEIII-F/R*	F: GGACCTAGAGTCCGCAAGAAGT R: CTACCCTTCACCTCTGCAGTGCT	Genomic fragment amplification
KASP	F: GAAGGTGACCAAGTTCATGCTACTGCTCTCCGCCGGTCGGCTGC**C** F: GAAGGTCGGAGTCAACGGATTACTGCTCTCCGCCGGTCGGCTGC**T** R: CCACAACTTCCTGCCAGGGGCCAC	KASP assay for SNP at 294 nt (C/T)
qRT-PCR-T-Allele qRT-PCR-C-Allele	F: GATGGGGACATGCCTAGCAATA R: CTGGACACGGGCTCCACCCAC F: GCCACGGTAACGCGTAACTGCT R: TCCGGACGGAGAAGACTATGCT	*TaSBEIII-A* expression in different tissues of wheat plants
qRT-PCR-TaActin	F: CTCCCTCACAACAACAACCGC	Control for endogenous
	R: TACCAGGAACTTCCATACCAAC	

### Total RNA Extraction

RNAprep pure Plant Kit was used for the extraction of RNA by following the instruction of the manufacturer. Similarly, by using FastQuant RT Kit (Tiangen Beijing), cDNA was synthesized.

### Expression Analysis

Two genotypes (Chinese Spring and Aikang-58) were planted on the basis of their *C* and *T* alleles in the field at the experimental station of the Institute of Crop Sciences, CAAS*Chinese Academy of Agricultural Sciences* with normal management. Samples were collected at different stages of both genotypes. The stages that were selected for sampling were leaves/shoot, seedling, spikes at the vegetative growth phase, grains at the reproductive phase (15 days after pollination), roots at the reproductive phase, leaves/shoot at the vegetative stage, spikes at the vegetative phase, spikes at the reproductive phase (after formation of spikes), and root seedling and roots at the vegetative growth phase.

One microgram of RNA was used to synthesize the cDNa with a TranScript Kit (TransGen, AT341) by following the manufacturer’s instructions. The concentration of cDNA was quantified with the help of NanoDrop and all the samples were made uniform for qRT-PCR. Two sets of primers were designed for qRT-PCR according to C and T alleles (Table 1). TransStart SuperMix Kit (TransGen, AQ131) was used for qRT-PCR and ran on a CFX96 system (Bio-Rad Co., United States). The process of amplification was initiated at 94°C for 30 s, followed by 43 cycles of denaturing for 5 s, annealing for 15 s, and extension for 10 s and then a melting curve stage. Both genotypes were used for qRT-PCR with three biological replications and three technical replications, and actin was used as a housekeeping gene for internal control. The relative expression value was calculated by Microsoft Excel.

### KASP Marker Development

On the variant of TaSBEIII-A, KASP primer was designed for high-throughput genotyping by following the standard KASP guidelines^2^ (Table 1). The specificity of primer was developed based on the standard FAM (5′-GAAGGTGACCAAGTTCATGCT-3′) and HEX (5′-GAAGGT CGGAGTCAACGGATT-3′) tails with a targeted SNP at the 3′ end. To check the diversity, the developed KASP marker was applied across the entire studied population. The methodology of KASP assay was followed as reported by Rehman et al. (2019). The clustering of accessions was shown on the scatter plot at the x (FAM) and y (HEX) signal.

### Association Analysis Between SNPs and Yield-Related Traits

Descriptive statistics and estimates of variance were done by using Microsoft Excel 2016. To check the effect of allelic variants on yield-related traits, Student’s *t*-test was used at *p* < 0.05 (even 0.01). The polymorphic information content (PIC) and gene diversity (*H*_e_) were measured online: https://www.gene-calc.pl/pic.

## Results

### Identification of Novel SNP in the CDS Sequence of *TaSBEIII-A*

PCR amplification and sequencing of *TaSBEIII-A* in 20 accessions allowed the identification of a SNP in the exon region of *TaSBEIII-A* at 294 bp. The transversion of SNP was “*C*” substituted into “*T*”. The SNP was non-synonymous and proline changed into serine amino acid. The alignment of sequences with Chinese Spring accession revealed that SNP is novel and was reported for the first time (Supplementary Figure 1). The sequence had been submitted to the NCBI GenBank and the accession number is MZ261926.

### High-Throughput KASP Marker for *TaSBEIII-A*

**FIGURE 2:**
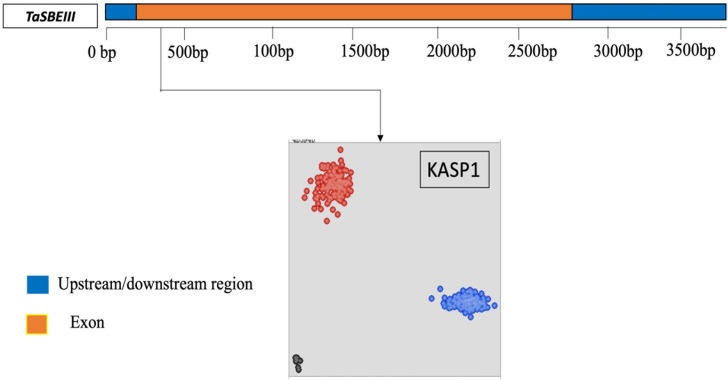
Gene structure and developed KASP marker for *TaSBEIII-A*. Gene structure of *TaSBEIII-A* consists of an upstream and downstream region with blue color and orange blocks representing the exon region. Bp represents the base pairs. The clusters of accessions are represented on the scatter plot on the *x*-axis (FAM) and *y*-axis (HEX). Accessions with blue color represent the FAM type of allele *C* while accession in red color denotes the Hex type of allele *T*. Black dots represent the non-template control (NTC).

A KASP marker was developed for the identified polymorphic site in the CDS sequence of *TaSBEIII-A* and named as a KASP1 marker. The frequency of this KASP1 marker was more than 5% in the MCC. In the scatter plot, the accession in blue circles have a “*C*” allele while the accessions in red circles have “*T*” alleles for the *TaSBEIII-A* gene (Figure 2). The polymorphism for this marker was identified in MCC, Pakistani wheat accessions, CIMMYT wheat accessions, and diploid and tetraploid wheat accessions (Figure 2).

### Association Between Allele and Yield-Related Ttraits

**FIGURE 3:**
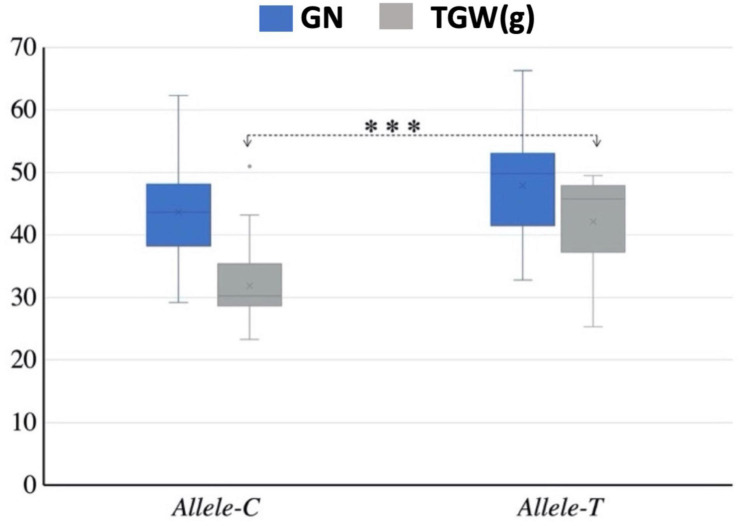
Interaction of favorable allele with yield-related traits. TGW, thousand grain weight; GN, grain number; *^∗^p* < 0.05, *^∗∗^p* < 0.01. The 2-year data of 2017 and 2018.

Mini core collection consisted of 262 accessions that were used to detect the association of *TaSBEIII-A* alleles with yield-related traits. Different yield-related traits data were collected such as TGW, number of grains per spike, spike length, and plant height. The data were collected with three replications and the average of these replications was used for association analysis. *Allele-T* was significantly associated with higher TGW (Figure 3). All other yield-related traits (spike length, number of grains per spike, and plant height) showed non-significant association between these two alleles. Therefore, on the basis of this result, it can be said that *Allele-T* significantly associated with yield-related traits and has the potential as a functional marker and that it can be used in MAS from improving starch content, which ultimately affects the yield of the grain.

### Expression Analysis of *TaSBEIII-A*

**FIGURE 4:**
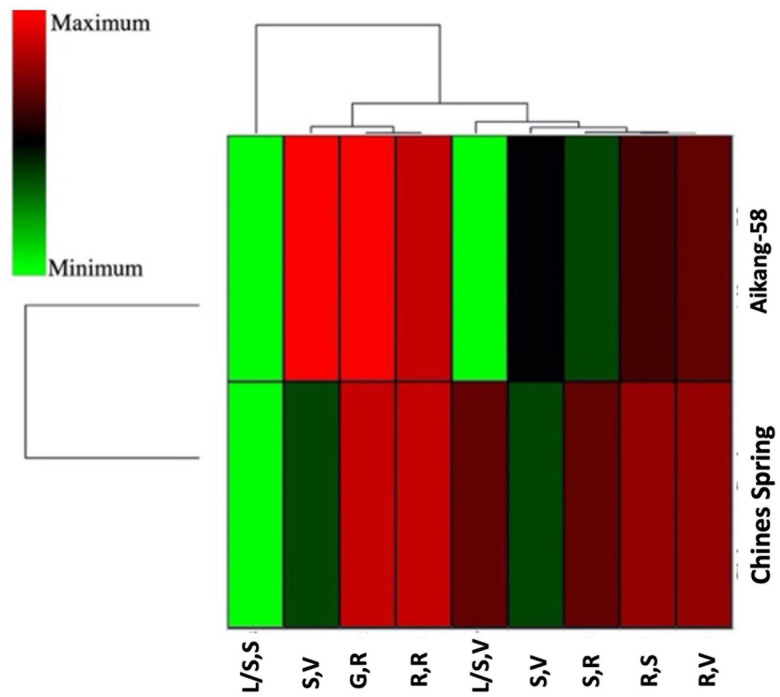
A heatmap of two genotypes having different alleles and their gene expression profile at different stages by using hierarchical clustering. XLSTAT was used to create a heatmap. R,S: root seedling; R,V: roots at the vegetative growth phase; R,R: roots at the reproductive phase; L/S,S: leaves/shoot seedling; L/S,V: leaves/shoot, vegetative; S,V: spikes at the vegetative growth phase; SR: spikes at the reproductive phase; GR: grains at the reproductive phase.

Two wheat accessions (Chinese Spring and Aikang-58) were selected based on allelic variation (*Allele-C* and *Allele-T*, respectively) to test the expression level of *TaSBEIII-A* in different stages of wheat plant. Primer specificity was checked on the basis of the melting curve of qRT-PCR for *TaSBEIII-A*, and *TaActin* gene was used as an internal control. qRT-PCR revealed that Aikang-58 with *Allele-T* had higher relative expression levels as compared with Chinese Spring having *Allele-C* at the spike reproductive stage and grain reproductive stage (Figure 4). These findings suggested that *Allele-T* might be responsible for high TGW and help to maintain a high starch content.

### PIC and Gene Diversity in Chinese and Non-Chinese Wheat Germplasm

To study the evolutionary history of *TaSBIII-A*, we analyzed *TaSBIII-A* in wheat ancestors. The results illustrated that during polyploidization events, diversity in *TaSBIII-A* increased. Diploid (AA) wheat accessions showed no diversity for *TaSBIII-A*. Tetraploid accessions (AABB) showed 0.3108 PIC and 0.3848 *H*_e_ for *TaSBIII-A*. PIC and *H*_e_ ranged between 0.3318 and 0.420 in hexaploid Chinese wheat landraces, respectively. For Chinese modern wheat cultivars, the PIC and *H*_e_ ranged between 0.3701 and 0.4902, respectively (Table 2). Among non-Chinese wheat germplasm, CIMMYT wheat accession showed 0.3749 PIC and 0.4998 *H*_e_, while Pakistani wheat accessions showed 0.3648 PIC and 0.48 *H*_e_.

**TABLE 2:** Polymorphic information content (PIC) and gene diversity (*H*_e_) values in studied wheat germplasms.

	**262MCC**	**CL**	**CM**	**CIMMYT**	**Tetra**	**PAK**
*H*_e_	0.455	0.42	0.4902	0.4998	0.3848	0.48
PIC	0.315	0.3318	0.3701	0.3749	0.3108	0.3648

*MCC, Mini core collection of China; CL, Chinese landraces; CM, Chinese modern cultivars; Tetra, Tetraploidy accessions; PAK, Pakistan.*

### Geographic Distribution of *TaSBEIII-A* Allelic Variation

**FIGURE 5:**
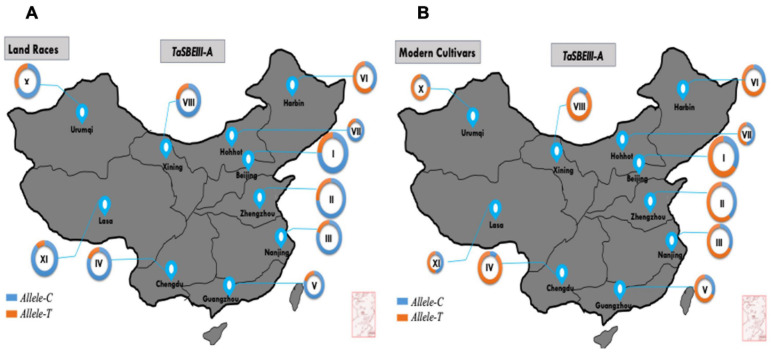
*TaSBEIII-A* alleles. Geographic distribution in China. **(A)** Landraces, **(B)** modern cultivars. I, Northern winter wheat zone; II, Yellow, and Huai River valleys winter wheat zone; III, Middle and low Yangtze valleys winter wheat zone; IV, Southwestern winter wheat zone; V, Southern winter wheat zone; VI, Northeastern spring wheat zone; VII, Northern spring wheat zone; VIII, Northwestern spring wheat zone; IX, Qinghai-Tibetan spring-winter wheat zone; X, Xinjiang winter-spring wheat zone. Pie chart size is directly proportional to the number of genotypes.

In China, wheat zones are divided on the basis of temperature, growing season, moisture contents, varietal response to photoperiod, and biotic and abiotic stress (Zhang et al., 2015). In this study, MCC was used to survey allelic variation of *TaSBEIII-A* in China, Pakistan, and CIMMYT wheat collections. Based on cultivation and production, zones I–IV are the major zones and cover 75% of the wheat area of China. The frequency of the favored allele *Allele-T* was lower in landraces and *Allele-C* was dominant in all the major zones of China. However, the frequency of favored *Allele-T* in modern cultivars was >50% in four major zones of wheat. The frequency of *Allele-T* showed a significant increment from 35 to 65% in zone I, 27 to 85% in zone II, 20 to 66% in zone III, and 19 to 85% in zone IV from landraces to modern cultivars, respectively (Figure 5). These results support the idea that favorable allelic variation was positively selected in all major zones and other zones of China with the passage of time. Therefore, this allele can be used further in breeding programs in China to increase the grain yield of wheat.

**FIGURE 6:**
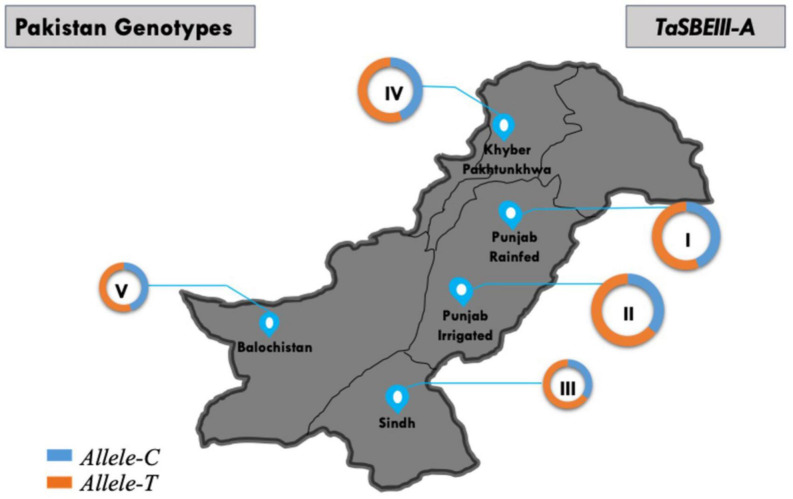
Distribution of *TaSBEIII-A* alleles among Pakistan accessions. I, Punjab rainfed zone; II, Punjab irrigated zone; III, Sindh region; IV, Balochistan zone; V, Khyber Pakhtunkhwa zone. Pie chart size is directly proportional to the number of accessions.

The geographic distribution of *TaSBEIII-A* was also investigated among Pakistani wheat accessions. The frequency of *Allele-T* was higher in Pakistan major zones such that its frequency was 64% in the Punjab irrigated zone and 55% in the Punjab rainfed zone. Similarly, the frequency in Khyber-Pakhtunkhwa was 65% and that in Sindh was 70%. There was a positive selection of the favorable allele in all zones of Pakistan (Figure 6). Similarly, in the CIMMYT germplasm, the frequency of *Allele-T* was higher than that of *Allele-C*.

### Positive Selection of *Allele-T* of *TaSBEIII-A* in Wheat Breeding History of China and Pakistan

**FIGURE 7:**
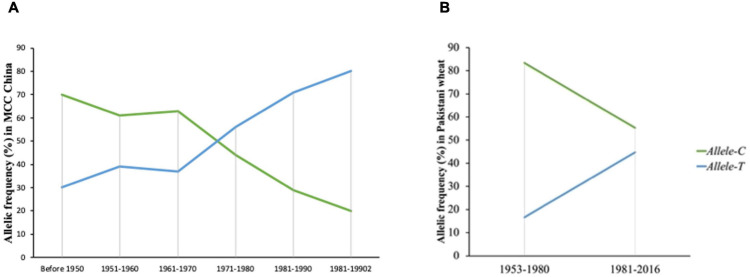
Selection of favored allele of *TaSBEIII-A* in wheat breeding history of China and Pakistan. **(A)** The frequency of China accessions. **(B)** The frequency of Pakistan accessions.

To evaluate the favorable allelic variation of *Allele-T*, MCC was used with known released dates and was divided into six groups (pre-1950, 1950s, 1960s, 1970s, 1980s, and 1990s). In general, accessions that were released before 1950 possessed *Allele-C* and few accessions had *Allele-T* (25%). The frequency of the favorable allele (*Allele-T*) increases from 1950 to 1960 (up to 38%) but remained stable from the 1960–1970 era. The frequency of the favorable allele increased from 1971 to 1990 (38 to 70%) and it became 80% in 2000 (Figure 7). From the 1960s onward, TGW also showed a continuous increasing trend (Supplementary Figure 2). These results indicated that this favorable variation is valuable and could be selected to further improve TGW in Chinese wheat germplasm.

Similar results had been depicted in the Pakistan accessions, which consist of 153 genotypes and divided into two groups (1953–1980 and 1981–2016). The two groups were determined on the basis of pre-green revolution (1953–1980) and post-green revolution (1981–2016). The initiation of green revolution started in Pakistan from the early 1970s. The frequency of *Allele-T* was 15% in 1953, and the frequency of *Allele-C* was 80%, but with passage, the frequency of the favorable allele increased. From 1981 to 2016, the frequency of the favorable allele was 55% (Figure 7). So, the favorable allele was also selected positively with the passage of time in Pakistani wheat accessions.

## Discussion

Due to domestication, evolution and breeding in wheat help to create a lot of genetic diversity in wheat germplasm. The level of polymorphism in wheat was 1 SNP/540 bp based on the bioinformatic analysis of large wheat EST database of 12 accessions (Somers et al., 2003). Similarly, genomic sequences consisting of coding and non-coding regions have 1 SNP/334 bp in the coding region and 1 SNP/267 bp in the genomic region (Ravel et al., 2006). In this study, *TaSBEIII* CDS was sequenced in the 20 diverse cultivars of wheat and polymorphism was detected. The SNP was detected in the exon region of *TaSBEIII-A* (Figure 2). There was no other SNP detected in the CDS sequence in the rest of the sub-genomes (B and D sub-genomes) for *TaSBEIII*. These results suggested that *TaSBEIII* is a conserved gene during evolution. Wheat D genome had a narrow genetic background with a lower level of polymorphism. This might be due to the fact that no SNPs in the CDS sequence of these sub-genomes have been reported yet (Rasheed et al., 2018b). Allele fixation during domestication and low genetic diversity in the wheat panel can also be other reasons for less polymorphism in this gene. There is a need to sequence diverse wheat germplasm to investigate these probabilities.

According to different research, it has been predicted that in wheat, per year genetic gain is ∼0.8 to 1% (Shearman et al., 2005; Zhou et al., 2007). Grain number per square meter plays a significant role for achieving genetic gain with a slight change in the grain weight (Gaju et al., 2009; Zhang et al., 2015). In this study, a SNP was identified for *TaSBEIII-A* in the CDS sequence of gene at 294 bp position and showed a significant association with TGW, which might be beneficial for improving grain yield. The expression of *TaSBEIII* was consistent at the grain filling stage, and the function of this gene might be different from other *SBE* genes (*SBEI*, *SBEIIa*, and *SBEIIb*) and might help to improve wheat yield. The function of this gene may be associated with the formation of A and B starch granules in the grains of wheat plant, which ultimately help in the wheat yield (Kang et al., 2013). Similarly in wheat, an open excess browser^3^ was developed to check the expression of the gene at different stages. The expression of this gene is also observed at the grain stage by using this web browser, which supports the results that show its function in grains of wheat (Borrill et al., 2016). The final dry weight of the grain contains 65 to 80% starch (Hurkman et al., 2003). The endosperm works as a storage tissue and contributes significantly to the yield of the grain. Therefore, the differential effects of the *TaSBEIII-A* allele on grain weight detected in the present study might be caused by different contributions to starch biosynthesis and hence to endosperm development. Generally, all over the world, a higher grain weight is the main objective of wheat breeders (Zhou et al., 2007).

The development of new genetic tools promises to address the challenges by improving the genetic gains of different crops and help to meet the world’s food production demand. The use of functional markers in wheat breeding programs through MAS is a successful approach to increase yield (Liu et al., 2014; Rasheed et al., 2018a). Recently the concept of MAS has been changed by shifting to whole-genome methods to attain maximum genetic gains in wheat breeding by using different complex traits (Zhao et al., 2014). The KASP1 marker was developed based on the SNP present at 294 bp in the CDS sequence of *TaSBEIII-A* (Figure 3). Two allelic variations (*Allele-C* and *Allele-T*) were observed in different wheat populations by using this KASP marker. By using further association analysis of these alleles, it was observed that *Allele-T* showed a significant association with high TGW in MCC. Based on this, it is depicted that this molecular marker can be instrumental in MAS to improve the yield of the wheat.

Polymorphic information content helps to understand the detailed knowledge of the level of polymorphism between accessions. On the basis of previous reports, PIC can be divided into three categories: (1) the marker is considered to highly polymorphic if the value is more than 0.5; (2) similarly, values between 0.25 and 0.4 indicate that the marker is moderately informative; and (3) the marker with a 0.25 PIC value is a low informative marker. In the present study, the average PIC value for landrace and modern cultivars was 0.32, while the value was increasing from tetraploid to hexaploid wheat accessions. This value is in agreement with previous studies using bi-allelic markers such as SNP or DArT in either common or durum wheat. In common wheat, a PIC value of 0.24 has been found for the WAMI population genotyped with the 9K SNP array (Rasheed et al., 2018a).

The favorable allele was also investigated in landraces and modern cultivars in China. The selection of the favorable allele of *TaSBEIII-A* showed an increasing trend from landraces to modern cultivars in wheat breeding in China. In major wheat-producing areas of China (Zones I, II, III, and IV), the frequency of the favorable *Allele-T* showed higher trends. Zones I, II, and III contribute about 64% of the total national area of China (Irshad et al., 2019), and accessions in these zones have a higher frequency of favorable alleles. The average grain weight in these regions is 42–44 g, especially in zone II (Barrero et al., 2011). Wheat yield mainly depends on the increase in TGW (Zhang et al., 2012). The frequency of favorable alleles increased from 1960 onward, and during that time, Chinese wheat varieties experienced a boom in their yield. In China, the main objective was to increase TGW in wheat varieties before 1960 (Wang et al., 2019). With the passage of time from 1970 to 1990, the breeding objective was also changed by adding other traits such as plant height, grain number per spike, and quality traits to improve the yield of Chinese wheat accessions (He et al., 2018). The change in breeding objective from 1950 to 1980 assisted in the positive selection of favorable alleles in Chinese wheat with a rapid increase in TGW before 1980 in wheat accessions (Figure 6). The frequency of favorable alleles increases by about 80% from the 1970s to the 1990s with an increase in TGW, which may be the reason for selecting the other favorable genes (Wang et al., 2018). Additionally, the frequency of favorable alleles increases in all 10 zones of China from landraces to modern cultivars. So, it can be said that this allele has large potential to increase TGW, which ultimately increases the yield of wheat crop.

Wheat accessions from Pakistan were also selected to evaluate the favorable allele diversity in the different wheat zones. In all major zones of Pakistan, the favorable allele was positively selected and with high frequency. From 1953 to 2016, the favorable allele was positively selected with the passage of time, showing that there is a positive selection of the favorable allele in the wheat breeding program of Pakistan. The population structure of Pakistan and China is different, but the positive selection of alleles in both germplasms is likely due to the high linkage disequilibrium of wheat in major yield genes, and these genes were elected during selection breeding (Semagn et al., 2014; Rossi et al., 2015; Irshad et al., 2019; Rehman et al., 2019). To confirm these results, there is a need to analyze this favorable allele in other wheat cultivars such as Europe, United States, and Australia. Based on the frequency result of these regions, the conclusion can be made that the selection of the favorable allele of *TaSBEIII-A* is due to the major yield-related genes.

In conclusion, high-throughput genotyping for MAS is of great importance. The molecular marker that was developed on the SNP *TaSBEIII-A* at 294 bp in which *Allele-T* was significantly associated with TGW and the frequency of this favorable allele increased about 80% from 1960 to 1990 in Chinese MCC. This allele can be used in future studies as selection criteria for improving yield traits. Thus, it is depicted that favorable alleles are valuable and could be selected to increase grain yield, and a gel-free KASP marker approach can help to improve the speed of wheat breeding.

## Data Availability Statement

The datasets presented in this study can be found in the NCBI Repository, accession number MZ261926 (https://www.ncbi.nlm.nih.gov).

## Author Contributions

AI, HG, JG, and LZ conceptualized the study. AI, SU, HG, XW, JG, HX, and CW performed the experiments and analyzed the data. AI, YX, LZ, SZ, and HG wrote the manuscript. LL reviewed the manuscript and assisted in the completion of the experiments. All authors contributed to the article and approved the submitted version.

## Conflict of Interest

The authors declare that the research was conducted in the absence of any commercial or financial relationships that could be construed as a potential conflict of interest.

## Footnotes

1 https://www.megasoftware.net/

2 http://www.lgcgenomics.com

3 http://www.wheat-expression.com
