# Supplementary material for: Identification of Single Nucleotide Polymorphism in TaSBEIII and Development of KASP Marker Associated With Grain Weight in Wheat
Source: Front Genet. 2021 Jul 9;12:697294. doi: 10.3389/fgene.2021.697294 (PMC8299302; doi:10.3389/fgene.2021.697294)
Supplement: Supplementary file 2 [file Data_Sheet_1.PDF]

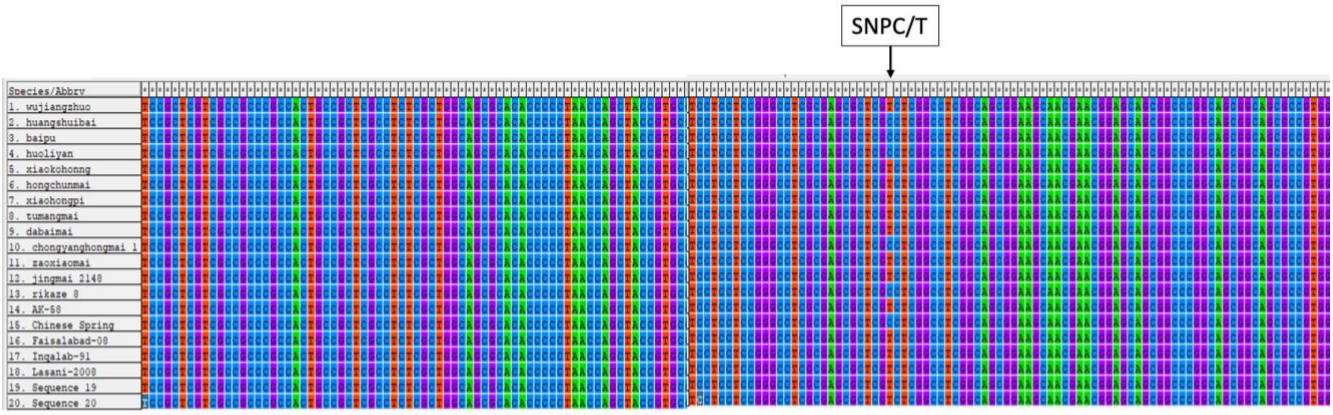

FIGURE S1. Alignment of twenty accession on MEGA software and identification of SNP.

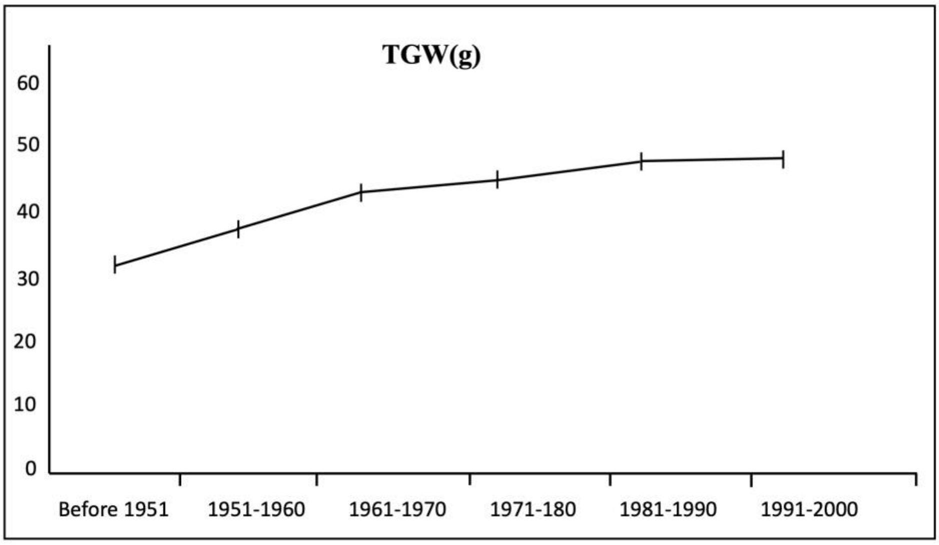

FIGURE S2. TGW changes from 1950 to 2000 in mini core collection of China. Error bars indicate the standard error.
